# Ethnomedicinal plants used by traditional healers in Phatthalung Province, Peninsular Thailand

**DOI:** 10.1186/s13002-015-0031-5

**Published:** 2015-05-30

**Authors:** Katesarin Maneenoon, Chuanchom Khuniad, Yaowalak Teanuan, Nisachon Saedan, Supatra Prom-in, Nitiphol Rukleng, Watid Kongpool, Phongsura Pinsook, Winyu Wongwiwat

**Affiliations:** Faculty of Traditional Thai Medicine, Prince of Songkla University, Songkhla, 90110 Thailand; Faculty of Health and Sports Science, Thaksin University, Phatthalung, 93110 Thailand; Faculty of Science and Technology, Songkhla Rajabhat University, Songkhla, 90000 Thailand

**Keywords:** Medicinal plants, Traditional healers, Peninsular Thailand, Phatthalung, Traditional knowledge

## Abstract

**Background:**

In rural communities of Thailand, traditional healers still play an important role in local health care systems even though modern medicine is easily accessible. Meanwhile, natural forests in Thailand which are important sources of materia medica are being greatly destroyed. This has led to an erosion of traditional Thai medicine. Furthermore, the concept of medicinal plant selection as medicine based on their tastes is still an important component of traditional Thai medicine, but no or little publications have been reported. Thus the aim of the present study is to collect ethnomedicinal data, medicinal plant tastes and relevant information from experienced traditional healers before they are lost.

**Methods:**

An ethnobotanical survey was carried out to collect information from nine experienced traditional healers on the utilization of medicinal plants in Phatthalung Province, Peninsular Thailand. Data were obtained using semi-structured interviews and participant observations. Plant specimens were also collected and identified according to the plant taxonomic method.

**Results:**

A total of 151 medicinal plants were documented and 98 of these are reported in the study. Local names, medicinal uses, parts used, modes of preparation, and the relationship between ailments and tastes of medicinal plant species are presented.

**Conclusions:**

This research suggests that traditional healers are still considered important for public health among Thai communities and that many people trust the healing properties of medicinal plants. In the future, it is hoped that traditional Thai medicine will be promoted and therefore will help reduce national public health expense.

## Background

Thailand has its own healing system of traditional medicine commonly referred to as “traditional Thai medicine”. This system is deeply rooted, and has played a key role in Thai culture for many centuries. The diverse way of life and culture in each separate region of Thailand has led to a diverse local health care system. This medicine depends on the knowledge and practical experience of each individual healer with regard to diagnosing and treating ailments using naturally available materials. Nowadays, Thai traditional medicine is supported by the government. It has been incorporated into national health policy for reducing the use of Western medicine which is very expensive. In 2012, the Ministry of Public Health wanted its subdistrict-level medical facilities to make traditional medicine account for 10 % of their total costs of medicine. Meanwhile, increasing the use of traditional medicine in community hospitals should account for 5 %. Moreover, at least one doctor who specializes in traditional medicine will work at community hospitals [1]. To respond to the government policy and develop the body of knowledge of Thai traditional medicine by using scientific approaches, many academic institutes have set up a 4-year program and curriculum for producing graduates with a Bachelor’s degree. However, there are many crucial issues concerning the transfer of knowledge of traditional medicine which is continually declining. Firstly, highly experienced traditional healers are generally older people and they continue to pass away without recording or passing on their knowledge. Secondly, the younger generation of medical practitioners has a low regard for traditional medicine. They are drawn to other occupations because of the job security and higher salaries. Finally, the forests in Thailand are being destroyed and this means that the medicinal plants necessary for traditional healers are in short supply. Consequently, the knowledge of the traditional healers regarding utilization of medicinal plants is being diminished and could possibly be lost before being explored by systematic study. The present study was carried out to document the diagnosis of diseases in general, details of the utilization of medicinal plants and the criteria for selecting medicinal plants in terms of taste property of traditional healers. The present study is the first research carried out in this area and it shows the correlation between medicinal plant tastes and plant selection for making an effective prescription which has never been reported before. Additionally, this ethnomedicinal information was collected from nine highly experienced traditional healers and it will directly benefit people who are interested in traditional medicine and medicinal plant aspects.

## Materials and methods

### Brief introduction to the study area

**Fig. 1:**
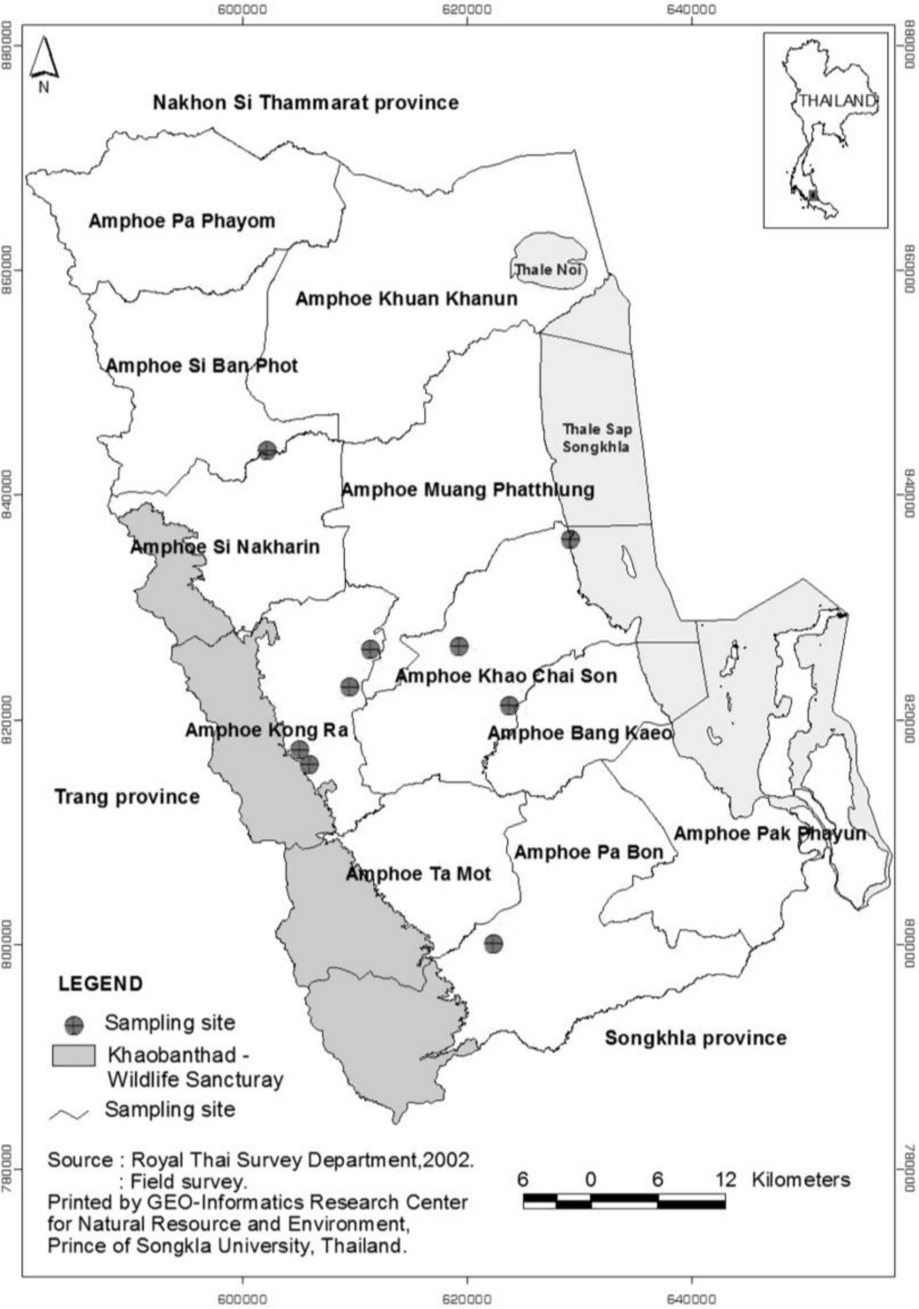
Location of the study sites in Phatthalung province, peninsular Thailand

Phatthalung is situated in southern Thailand. It is geographically located between latitude: 7° 05 to 7° 55 N and longitude: 99° 44 to 100° 25 E. The total area is 3424.473 km^2^. Most areas in Phatthalung can be classified into one of two classifications: 1) the eastern part is flood plain and some rolling terrain with an elevation that ranges between 0–15 m above sea level, 2) the western part is mountainous terrain covered by evergreen forest with an elevation that ranges between 50–1200 m above sea level [2]. It borders Nakhon Si Thammarat province to the north, Songkhla province to the south, Songkhla Lake to the east and Khaobanthad wildlife sanctuary to the west, which is covered with rich evergreen forest (Fig. 1). The annual average rainfall is 1800 mm. Rainfall distribution is divided into 2 periods, the long rains from September to January and the short rains from May to June. The average annual temperature is 28 °C [3]. Phatthalung is divided into 11 districts, Bang Kaeo, Khao Chaison, Khuan Khanun, Kong Ra, Mueang, Pa Bon, Pa Phayom, Pak Phayun, Si Banphot, Srinagarinda and Tamot. The population was approximately 514,492 in the year 2012. Most of the population is Buddhist followed by Muslim. Most people live in rural areas and their main occupation is in agriculture. The agriculture types include rubber, rice, pineapple, fruit orchards, cattle, poultry and fisheries. The way of life of the people still depends on natural products in their daily lives. Although the modern health care system is easy to access, many people still believe in traditional medicine. Therefore, traditional healers are important people in the communities. In the study area, there were various types of traditional healers such as herbalists, spiritual healers, midwives, massage practitioners and bone healers. The present study was focused only on herbalists who resided in rural areas.

### Ethnobotanical data collection

The ethnobotanical survey was carried out in Phatthalung province from 2010 to 2012. With an emphasis on accurate information, different types of ethnographic method, such as semi-structured interviews, participatory observation and fieldnotes, were combined to collect data with traditional healers from different areas of Phatthalung province, peninsular Thailand. Before starting the ethnobotanical data collection, snowball sampling was used as a practical method for finding highly experienced traditional healers from Phatthalung provincial health office. Patients who were cured by traditional healers were sought, along with community leaders and also traditional healers who had a well-established network. All traditional healers were selected not only for their extensive experience of traditional treatments, but also because they still actively practised their treatment with patients, including highly respected people within the communities. According to intensive criteria, nine highly experienced traditional healers were chosen. They were all males and their ages ranged from 55 to 110. Their experience ranged from 20 to 70 years in the field of traditional medicine. Five were Muslim and the rest were Buddhist. In terms of educational level, five had attended primary school and four had attended high school. The majority worked in agricultural professions. Their knowledge of traditional medicine was inherited from ancestors and close relatives, and additionally they studied from other experienced traditional healers. The main method of healing was by using medicinal plants. Only one traditional healer treated an ailment with rituals if he thought that an ailment was caused by supernatural forces. The interviews sought to determine the vernacular name of the plant, purposes of utilization, parts used, diseases treated, modes of preparation, administration and taste of individual plants as well as places of collection. Before interviewing commenced, the aims of the study were clearly explained to traditional healers and their family members. Prior informed consent was obtained. A copy of the final report of the study was sent to all traditional healers. The accuracy of information was rechecked by repeatedly visiting all healers at least 4–5 times in different seasons. Observing their various activities and staying at their homes during data collection was also necessary. The interviews were supplemented by walking in the field with traditional healers while collecting plants and checking the habitat preference of plants. The plant specimens were photographed, collected and processed according to the plant taxonomic method [4]. The specimens were identified and the voucher specimens collected from the wild were deposited at the PSU Herbarium. In addition, cultivated and common species were deposited at the herbarium within the Traditional Thai Medicine Faculty, Prince of Songkla University. To analyze the utilization of medicinal plants they were divided into groups of diseases based on properties and applications that were mentioned by the traditional healers.

## Results and discussion

### Diagnostic methods

Based on the study, all traditional healers indicated that illness is caused by the imbalance of the four body elements, soil, water, wind and fire, known as “Tard chao ruan” in Thai. For precise illness diagnosis, several procedures are integrated such as checking the patient’s medical history, physical examinations and pulse - taking. The principal history of patients includes behavioral issues, such as consumption, sleepiness and bowel movements. Feeling body parts is important for physical examination, as is close observation of the skin, eyes, tongue and hair. The taking of the pulse is common with highly experienced traditional healers. They say that they look for the duration between pulses. Different pulse characteristics are used to determine the deficiency of body elements and levels of severity. This procedure is very important and if the traditional healer has sufficient skill, it can give a precise diagnosis. However, very few traditional healers have the necessary pulse - taking skills, especially among the younger generation. After they have ascertained the cause of the illness, traditional prescriptions and treatments are assigned. Prescribing the correct use of medicinal plants is another important skill, which is explained further in the section of this document about medicinal plant tastes.

### Diversity of medicinal plants

**Fig. 2:**
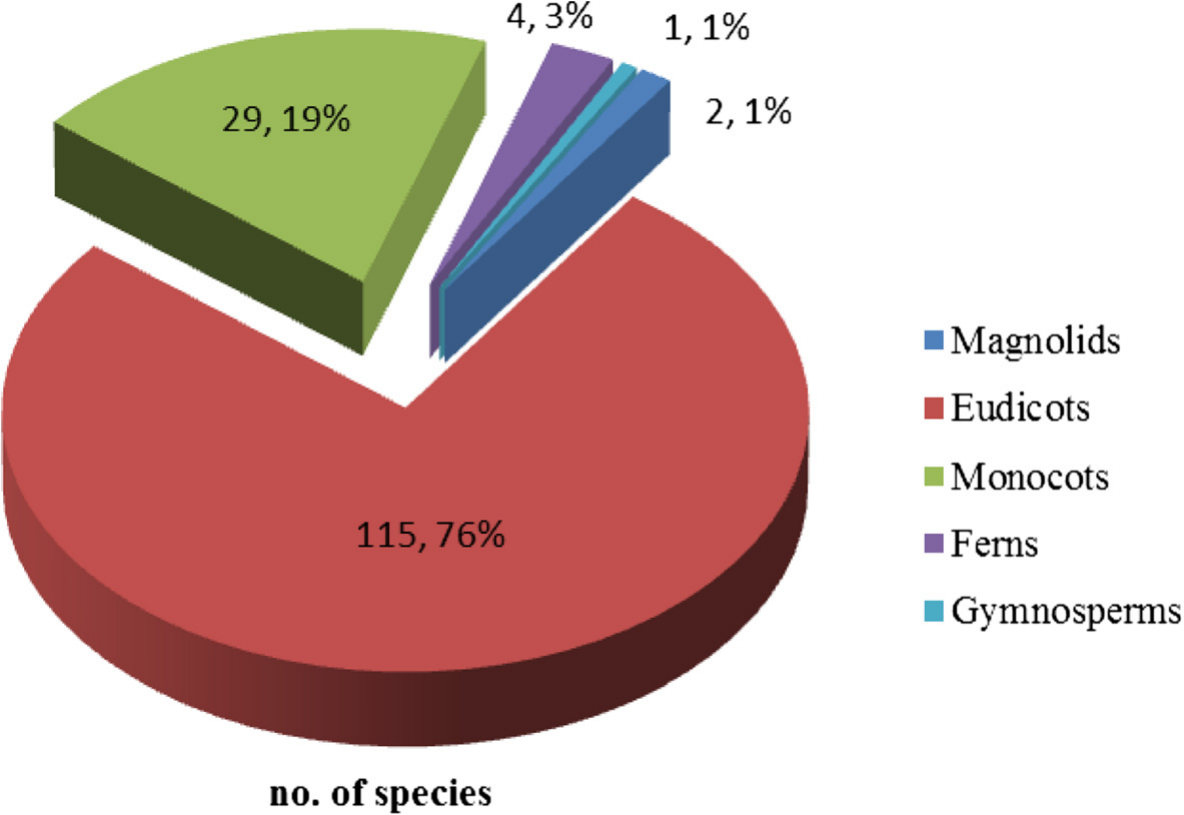
Groups of medicinal plants

According to the study, 151 species of medicinal plants, belonging to 126 genera in 60 families were documented for various disease treatments. The largest number of medicinal plants were eudicots (76 %) followed by monocots (19 %), ferns (3 %) and gymnosperms and magnoliids (1 % each) (Fig. 2). Of the eudicots, the most represented family was Fabaceae (nine species), followed by Lamiaceae (six species). The most dominant family of monocots was Zingiberaceae (ten species). Other families with low numbers included 30 families which represented only one species. This result was in agreement with the previous study, which mentioned that the most dominant family was Fabaceae [5–7]. As documented here, 98 species were presented which were cited by more than half of the traditional healers (Table 1).

**Table 1:** List of major uses of medicinal plants cited by more than five traditional healers

Scientific name/voucher number	Local name	Taste	Ailments treated / uses	Parts used / form of administration
Magnoliids				
Annonaceae				
1. *Goniothalamus macrophyllus* (Blume) Hook. f. & Thomson (N. Saedan & C. Khuniad 096)	Chingdokdiao	hot	alleviates body pain, flatulence, suffers from general fatigue	root / decoction / int
Piperaceae				
2. *Piper chaba* Hunt (Y. Teanuan & S. Prom- In 099)	Di pli	hot	flatulence, strengthens the body elements	fruit / decoction / int
3. *Piper sarmentosum* Roxb. (Y. Teanuan & S. Prom- In 048)	Cha phlu	hot	flatulence, strengthens the body elements, paralysis	root / decoction / int
Eudicots				
Acanthaceae				
4. *Andrographis paniculata* (Burm. f.) Wall. ex Nees/(N. Saedan & C. Khuniad 008)	Fa thalai chon	bitter	fever	whole plant/juice, decoction/int
5. *Clinacanthus nutans* (Burm. f.) Lindau (N. Saedan & C. Khuniad 006)	Phaya yo	bland	poisonous animal bites, antidote, canker	leaf/ juice, decoction / ext
6. *Justicia adhatoda* L. (Y. Teanuan & S. Prom- In 024)	Sa niat	bitter	eliminates sputum, cough, fever	leaf /juice, decoction / int
7. *Rhinacanthus nasutus* (L.) Kurz (N. Saedan & C. Khuniad 004)	Thongphanchang	nauseating	skin diseases, eczema, scabies	leaf/ paste / ext
8. *Thunbergia laurifolia* Lindl.	Rang chuet	bland	antidote, skin irritation	leaf / juice / int, ext
Amaranthaceae				
9. *Alternanthera bettzickiana* (Regel) G. Nicholson (Y. Teanuan & S. Prom- In 090)	Phak pet daeng	bland	arthritis, improves blood circulation, emmenagogue, menstrual cramps	whole plant/ decoction / int
Apiaceae				
10. *Centella asiatica* (L.) Urb. (Y. Teanuan & S. Prom- In 063)	Bua bok	bitter	thirst, fever, skin swelling	whole plant / paste / int, ext
Asteraceae				
11. *Acmella oleracea* (L.) R. K. Jansen (Y. Teanuan & S. Prom- In 010)	Phak khrat hua wan	hot	toothache	fruit / juice / int
12. *Blumea balsamifera* (L.) DC. (N. Saedan & C. Khuniad 014)	Nat yai	aromatic	promotes blood flow, flatulence, itching	leaf/ decoction / int
13. *Chromolaena odoratum* (L.) R. M. King & H. Rob. (W. Wongwiwat & S. Pinsook 008)	Sap suea	nauseating	stops bleeding, peptic ulcer	leaf/paste, decoction /int, ext
14. *Eclipta prostrata* (L.) L. (Y. Teanuan & S. Prom- In 088)	Ka meng	nauseating	stops bleeding, skin diseases, cervical diseases	whole plant /paste, decoction / int
15. *Elephantopus scaber* L. (N. Saedan & C. Khuniad 097)	Do mai ru lom	bland	tonifies the muscles	whole plant / decoction / int
16. *Pluchea indica* (L.) Less. (Y. Teanuan & S. Prom- In 025)	Khlu	bland	diuretic	leaf/ decoction / int
17. *Vernonia cinerea* (L.) Less. (N. Saedan & C. Khuniad 009)	Ya dok khao	bland	smoking cessation, alleviates body pain, fever	whole plant /smoking, decoction / int
Capparidaceae				
18. *Capparis micracantha* DC. (Y. Teanuan & S. Prom- In 019)	Chingchi	bitter	fever, strengthens the body elements	root / decoction / int
Clusiaceae				
19. *Garcinia mangostana* L. (Y. Teanuan & S. Prom- In 020)	Mang khut	astringent	skin diseases, intestinal infection	pericarp / juice, decoction / ext, int
Cucurbitaceae				
20. *Coccinia grandis* (L.) Voigt (Y. Teanuan & S. Prom- In 111)	Tamlueng	bland	poisonous animal bites, fever	leaf / juice / ext
21. *Gymnopetalum chinense* (Lour.) Merr. (Y. Teanuan & S. Prom- In 094)	Kadom	bitter	fever, tonifies the liver	fruit / decoction / int
22. *Momordica charantia* L. (Y. Teanuan & S. Prom- In 095)	Mara khinok	bitter	fever, appetizer, tonifies the liver	fruit / decoction / int
Euphorbiaceae				
23. *Croton roxburghii* N. P. Balakr. (Y. Teanuan & S. Prom- In 102)	Plao yai	hot	alleviates body pain, flatulence, prevention of fever after parturition	wood / decoction / int
24. *Croton tiglium* L. (Y. Teanuan & S. Prom- In 071)	Salot	hot	diuretic, hemorrhoids, constipation	root, seed oil / decoction / int
25. *Croton stellatopilosus* Ohba (Y. Teanuan & S. Prom- In 011)	Plao noi	hot	flatulence, peptic ulcer, stomachache	wood / decoction / int
26. *Excoecaria oppositifolia* Griff. (N. Saedan & C. Khuniad 023)	Fai duean ha	hot	menstrual problems	wood / decoction / int
27. *Shirakiopsis indica* (Willd.) Esser (N. Saedan & C. Khuniad 025)	Samo thale	sour	constipation	fruit / decoction / int
Fabaceae				
28. *Abrus precatorius* L. (N. Saedan & C. Khuniad 026)	Ma klam khruea	sour	fever, sore throat	root / decoction / int
29. *Albizia myriophylla* Benth. (W. Wongwiwat & S. Pinsook 096)	Cha em thai	sweet	strengthens the body in general, sore throat	root, wood / decoction / int
30. *Cassia fistula* L. (Y. Teanuan & S. Prom- In 100)	Ratcha phruek	sweet	fever, constipation, eliminates sputum (mixed with bitter salt for constipation)	fruit, pulp / decoction / int
31. *Caesalpinia major* (Medik.) Dandy & Exell (W. Wongwiwat & S. Pinsook 126)	Sa wat	nauseating	expels worms	leaf / decoction / int
32. *Derris scandens* (Roxb.) Benth. (N. Saedan & C. Khuniad 027)	Thaowanpriang	nauseating	alleviates body pain	wood / decoction / int
33. *Mimosa pudica* L. (Y. Teanuan & S. Prom- In 014)	Maiyarap	bland	fever, measles, chickenpox	whole plant / decoction / int
34. *Senna alata* (L.) Roxb. (N. Saedan & C. Khuniad 028)	Chumhet thet	nauseating	skin diseases, constipation	leaf / paste / ext; inflorescence / decoction / int
35. *Senna tora* (L.) Roxb. (Y. Teanuan & S. Prom- In 029)	Chumhet thai	nauseating	constipation, skin diseases, appetizer, insomnia	whole plant / decoction / int
36. *Tamarindus indica* L. (Y. Teanuan & S. Prom- In 147)	Ma kham	sour	eliminates sputum, improves blood flow, constipation, cold in children	fruit / decoction / int
Gentianaceae				
37. *Fagraea fragrans* Roxb.	Kan krao	bitter	fever	wood / decoction / int
Lamiaceae				
38. *Clerodendrum petasites* (Lour.) S. Moore (Y. Teanuan & S. Prom- In 137)	Mai thoa yai mom	bitter	fever, constipation	root / decoction / int
39. *Clerodendrum serratum* (L.) Moon (N. Saedan & C. Khuniad 022)	Ak khi thawan	bitter	hemorrhoids, itching	leaf / decoction / int
40. *Ocimum americanum* L. (Y. Teanuan & S. Prom- In 116)	Mang lak	aromatic	flatulence, increases milk production	leaf / decoction / int
41. *Ocimum basilicum* L. (Y. Teanuan & S. Prom- In 117)	Horapha	aromatic	flatulence, increases milk production	leaf / decoction / int
42. *Ocimum tenuiflorum* L. (Y. Teanuan & S. Prom- In 115)	Ka phrao daeng	aromatic	flatulence, dizziness, asthma in children	leaf / decoction / int, ext
43. *Orthosiphon aristatus* (Blume) Miq. (Y. Teanuan & S. Prom- In 152)	Ya nuat maeo	bland	diuretic	whole plant / decoction / int
Lythraceae				
44. *Punica granatum* L. (Y. Teanuan & S. Prom- In 139)	Thap thim	astringent	dysentery, diarrhea	pericarp / decoction / int
Malvaceae				
45. *Sida rhombifolia* L. (Y. Teanuan & S. Prom- In 028)	Khat mon	fat	severe fever, liver diseases, alleviates body pain	whole plant / decoction / int
Menispermaceae				
46. *Tiliacora triandra* (Colebr.) Diels (Y. Teanuan & S. Prom- In 092)	Yanang	bland	fever, antidote	root / decoction / int
47. *Tinospora crispa* (L.) Miers ex Hook. f. & Thomson (N. Saedan & C. Khuniad 016)	Bora phet	bitter	promotes blood flow, appetizer, fever, diabetes, reduces body heat, thirst	wood / decoction / int
Opiliaceae				
48. *Lepionurus sylvestris* Blume (Y. Teanuan & S. Prom- In 018)	Mak mok	fat	strengthens the body in general, promotes blood flow, alleviates body pain, cervical problems	root / decoction / int
Phyllanthaceae				
49. *Bridelia ovata* Decne. (Y. Teanuan & S. Prom- In 076)	Maka	bitter	constipation	leaf / decoction /int
50. *Phyllanthus emblica* L. (N. Saedan & C. Khuniad 015)	Ma kham pom	sour	promotes blood flow, eliminates sputum, cough, thirst, mixed with *Terminalia chebula* Retz. and *Terminalia bellirica* (Gaertn.) Roxb. for body tonic (Tri pha la)	fruit / decoction / int
51. *Phyllanthus pulcher* Wall. ex MÜll. Arg. (W. Wongwiwat & S. Pinsook 094)	Thorani san	bland	fever	root/ decoction / int
52. *Phyllanthus urinaria* L.	Ya tai bai	bitter	alleviates body pain, fever	whole plant / decoction / int
53. *Sauropus androgynus* (L.) Merr. (Y. Teanuan & S. Prom- In 030)	Phak wan ban	bland	fever, increases milk production	leaf / decoction / in
Plumbaginaceae				
54. *Plumbago indica* L. (Y. Teanuan & S. Prom- In 120)	Chetta mun phloeng daeng	hot	strengthens the body elements, emmenagogue	root / decoction / int
55. *Plumbago zeylanica* L. (Y. Teanuan & S. Prom- In 073)	Chetta mun phloeng khao	hot	strengthens the body elements, emmenagogue, improves blood flow	root / decoction / int
Rubiaceae				
56. *Mitragyna speciosa* (Roxb.) Korth. (Y. Teanuan & S. Prom- In 012)	Kra thom	nauseating	dysentery, diarrhea, skin diseases	leaf / decoction / int/ext
57. *Morinda citrifolia* L. (Y. Teanuan & S. Prom- In 078)	Yo ban	aromatic	dizziness	fruit / decoction / int
58. *Morinda elliptica* Ridl. (W. Wongwiwat & S. Pinsook 024, 079)	Yo pa	hot	flatulence, women’s diseases	wood / decoction / int
59. *Saprosma brunneum* Craib (Y. Teanuan & S. Prom- In 046)	Phahom ton	aromatic	strengthens the body in general, flatulence	whole plant / decoction / int
Rutaceae				
60. *Aegle marmelos* (L.) Corrêa ex Roxb. (N. Saedan & C. Khuniad 110)	Matum	fat	strengthens the body elements	fruit / decoction / int
61. *Citrus aurantifolia* (Christm.) Swingle (Y. Teanuan & S. Prom- In 132)	Manao	sour	cough, eliminates sputum	fruit / juice / int
62. *Citrus hystrix* DC. (Y. Teanuan & S. Prom- In 131)	Makrut	aromatic	flatulence, dizziness, eliminates sputum, improves blood flow	pericarp / decoction / int
63. *Zanthoxylum* cf. *nitidum* (Roxb.) DC. (W. Wongwiwat & S. Pinsook 077)	Phakrut	aromatic	expels worms, diarrhea, toothache	wood / decoction / int
Sapindaceae				
64. *Cardiospermum halicacabum* L. (Y. Teanuan & S. Prom- In 042)	Khok kra om	bland	itching	whole plant / juice / ext
Sapotaceae				
65. *Mimusops elengi* L. (Y. Teanuan & S. Prom- In 043)	Phikun	aromatic	tonifies the heart, dizziness	flower / decoction / int
Simaroubaceae				
66. *Brucea javanica* (L.) Merr. (W. Wongwiwat & S. Pinsook 079)	Ratcha dat	bitter	malaria	whole plant / decoction / int
67. *Eurycoma longifolia* Jack (Y. Teanuan & S. Prom- In 111)	Pla lai phueak	bitter	alleviates body pain, fever, malaria	root / decoction / int
68. *Harrisonia perforata* (Blanco) Merr. (N. Saedan & C. Khuniad 037)	Khontha	bitter	fever	root / decoction / int
Solanaceae				
69. *Datura metel* L. var. *metel* (Y. Teanuan & S. Prom- In 130)	Lam phong	nauseating	skin diseases	leaf / paste / int
70. *Solanum indicum* L. (Y. Teanuan & S. Prom- In 149)	Mawaeng ton	bitter	cough, eliminates sputum	fruit / juice / int
71. *Solanum trilobatum* L. (N. Saedan & C. Khuniad 038)	Mawaeng khruea	bitter	cough, eliminates sputum	fruit / juice / int
Thymelaeaceae				
72. *Aquilaria malaccensis* Lam. (Y. Teanuan & S. Prom- In 001)	Kritsana	aromatic	tonifies heart, alleviates fatigue, promotes blood flow, fever, strengthens the body in general, dizziness	wood / decoction / int
Vitaceae				
73. *Cissus quadrangularis* L. (N. Saedan & C. Khuniad 003)	Phet sangkhat	nauseating	hemorrhoids, sinusitis, intestinal infection	wood / capsule / int
Monocots				
Acoraceae				
74. *Acorus calamus* L. (N. Saedan & C. Khuniad 039)	Wan nam	aromatic	flatulence, strengthens the body elements	rhizome / decoction / int
Araceae				
75. *Amorphophallus* cf. *paeoniifolius* (Dennst.) Nicolson (Y. Teanuan & S. Prom- In 057)	Buk	nauseating	habitual constipation, skin diseases	tuber / decoction / int
76. *Lasia spinosa* (L.) Thwaites (Y. Teanuan & S. Prom- In 057)	Phak nam	nauseating	habitual constipation, skin diseases	rhizome / decoction / int
Asparagaceae				
77. *Asparagus racemosus* Willd. (N. Saedan & C. Khuniad 041)	Sam sip	fat	strengthens the body in general	root / decoction / int
Asphodelaceae				
78. *Aloe vera* (L.) Burm. f. (N. Saedan & C. Khuniad 042)	Wan hang chora khe	bland	peptic ulcer, poisonous animal bites, burns	leaf / juice / int, ext
Cyperaceae				
79. *Cyperus rotundus* L. (Y. Teanuan & S. Prom- In 151)	Haeo mu	aromatic	flatulence, strengthens the body elements	corm / decoction / int
Dioscoreaceae				
80. *Dioscorea hispida* Dennst. (W. Wongwiwat & S. Pinsook 011)	Kloi	nauseating	tuber: strengthens the body in general; stem: sinusitis	tuber, stem / decoction / int
81. *Tacca chantrieri* Andrê (Y. Teanuan & S. Prom- In 017)	Khang khao dam	bland	sexual stimulants	rhizome / decoction / int
82. *Tacca integrifolia* Ker Gawl. (N. Saedan & C. Khuniad 052)	Wan nang khruan	bland	alleviates body pain, sexual stimulants	rhizome / decoction / int
Marantaceae				
83. *Donax grandis* (Miq.) Ridl. (Y. Teanuan & S. Prom- In 025)	Khlum	bland	fever	rhizome/ decoction / int
84. *Schumannianthus dichotomus* (Roxb.) Gagnep. (Y. Teanuan & S. Prom- In 018)	Khla	bland	skin diseases, fever, reduces body heat	rhizome / decoction / int
Poaceae				
85. *Panicum repens* L. (Y. Teanuan & S. Prom- In 021)	Ya khrun	bland	diuretic	whole plant / decoction / int
Stemonaceae				
86. *Stemona tuberosa* Lour. (W. Wongwiwat & S. Pinsook 078)	Non tai yak	nauseating	kill parasites	root / juice / int, ext
Zingiberaceae				
87. *Amomum testaceum* Ridl. (N. Saedan & C. Khuniad 045)	Krawan	aromatic	flatulence, promotes blood flow, asthma, menstrual problems	fruit / decoction / int
88. *Boesenbergia rotunda* (L.) Mansf. (Y. Teanuan & S. Prom- In 113)	Kra chai	aromatic	strengthens the body in general, increases milk production, dysentery	root / juice / int
89. *Curcuma comosa* Roxb. (Y. Teanuan & S. Prom- In 022)	Wan chak motluk	astringent	discharge amniotic fluid after giving birth, treat postpartum uterine swelling	fruit / decoction / int
90. *Curcuma longa* L. (Y. Teanuan & S. Prom- In 023)	Khamin chan	astringent	peptic ulcer, intestinal infections, skin diseases	rhizome / paste / ext, int
91. *Curcuma zedoaria* (Berg) Roscoe (Y. Teanuan & S. Prom- In 114)	Khamin oi	astringent	skin diseases	rhizome / paste / ext
92. *Kaempferia galanga* L. (W. Wongwiwat & S. Pinsook 028)	Pro hom	aromatic	flatulence	rhizome / juice / int
93. *Kaempferia parviflora* Wall. ex. Baker (N. Saedan & C. Khuniad 046)	Krachai dam	aromatic	strengthens the body in general, sexual stimulants	rhizome / extracted with alc. / int
94. *Zingiber montanum* (Koenig) Link ex Dietr. (Y. Teanuan & S. Prom- In 040)	Phlai	astringent	flatulence, alleviates muscle pain, strain, skin swelling (mixed with “Ya dam”, latex of *Aloe vera* L.)	rhizome / juice, decoction / int
95. *Zingiber officinale* Roscoe (Y. Teanuan & S. Prom- In 062)	Khing	hot	flatulence, appetizer	rhizome / juice, decoction / int
96. *Zingiber zerumbet* (L.) Sm. (Y. Teanuan & S. Prom- In 122)	Kra thue	bitter	flatulence, dysentery	rhizome / juice, decoction / int
Gymnosperms				
97. *Gnetum montanum* Markgr. (N. Saedan & C. Khuniad 071)	Ma mueai	nauseating	alleviates muscle pain	wood / decoction / int
Ferns				
Polypodiaceae				
98. *Drynaria quercifolia* (L.) Sm. (N. Saedan & C. Khuniad 032)	Kratae tai mai	bland	diuretic	rhizome / decoction / int

**Fig. 3:**
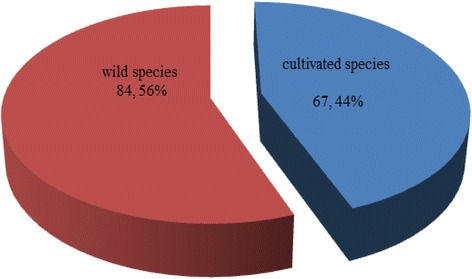
Sources of medicinal plants

**Fig. 4:**
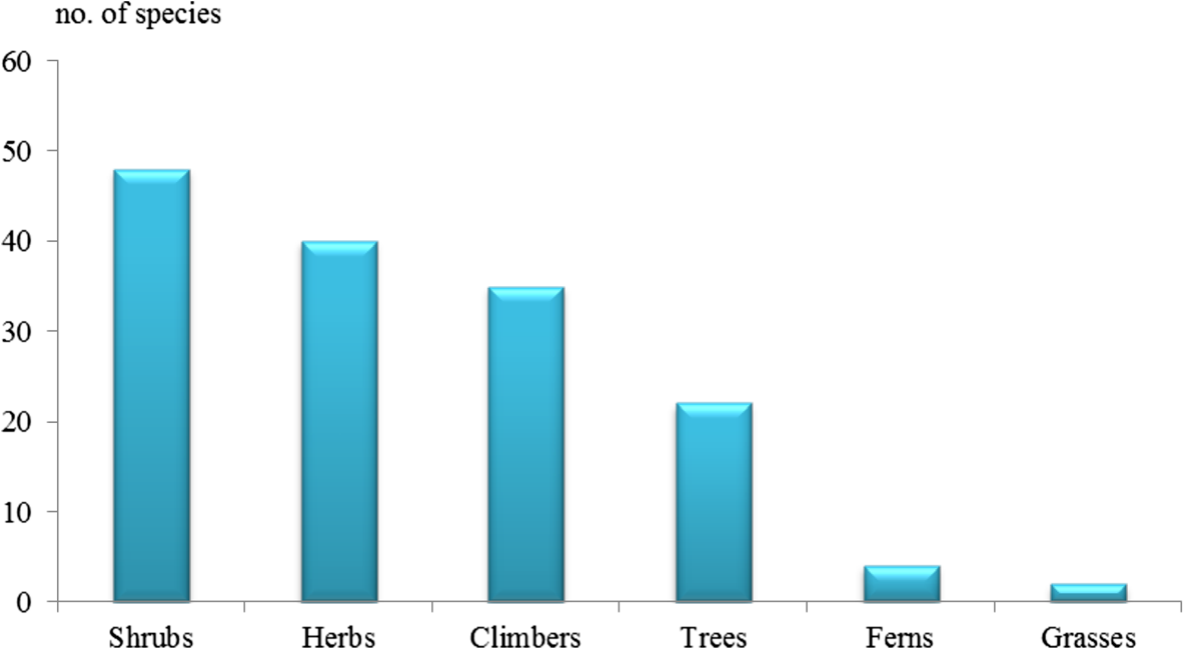
Habits of medicinal plants

The result of this study indicated that the majority of medicinal plants used by traditional healers are still harvested from the wild (Fig. 3). In the case of cultivated plants, the original habitats were wild and located far away from the traditional healers’ villages. For convenience, these medicinal plants were moved and planted in cultivated fields or home gardens and used whenever required. However, most traditional healers said that the current situation of medicinal plants is a concern. Some medicinal plant species have become rare or extinct because of overexploitation and continued deforestation. As a result, the shortage of medicinal plants has affected healing treatments. The result was in agreement with the study of Tabuti [7] and Wodah and Asase [8] who reported on the decrease of medicinal plants in northwest Ghana and Uganda. This study showed that shrubs were found to be the most used plants (48 species) followed by herbs (40 species), climbers (35 species) and trees (22 species) (Fig. 4). This result indicated that shrubs were common and easily harvested when compared to others. Furthermore, most of the study areas for this study were open areas suitable for shrub growing.

### Plant parts used

**Fig. 5:**
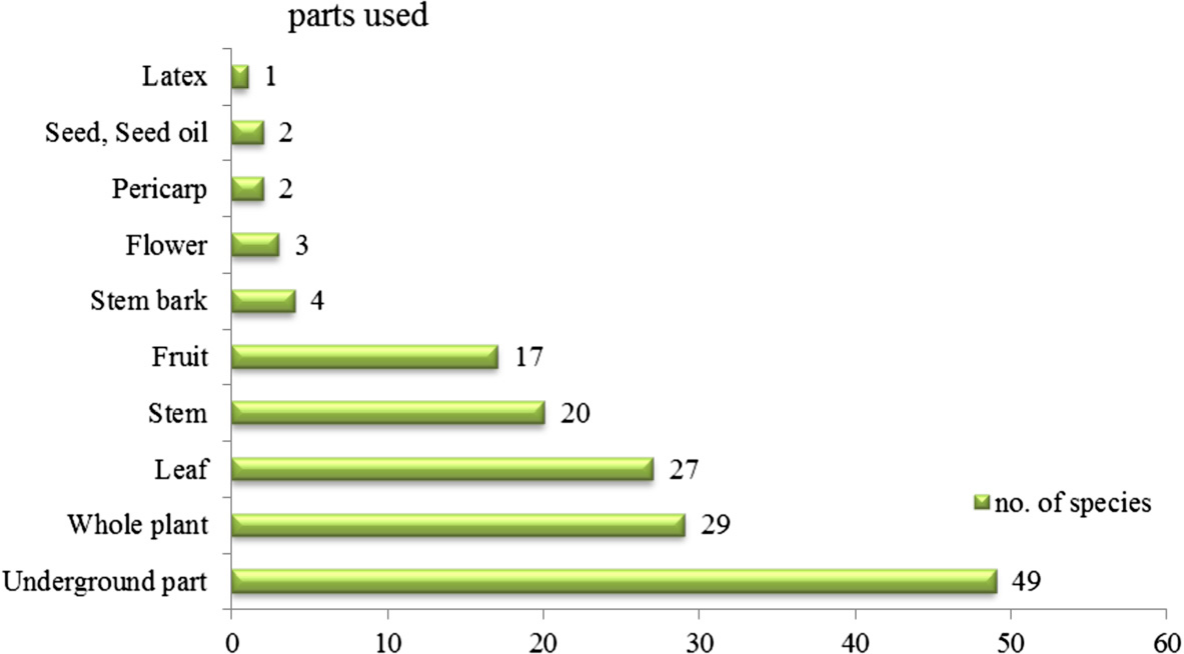
Parts used of medicinal plants

Among the different parts of medicinal plants used by traditional healers, the underground parts (root, rhizome, tuber, corm) were most frequently used to make the prescriptions for healing treatments, while the whole plant and leaves were second and third respectively (Fig. 5). The study was in agreement with the study of Tabuti [7], Cheikhyoussef [5] and Wodah and Asase [8]. Interestingly, Tabuti [7] mentioned that the uses of root and tuber parts can threaten medicinal plant populations or species viability. This observation was in agreement with this study, most traditional healers said that some species such as *Goniothalamus macrophyllus* (Blume) Hook. f. & Thomson, *Capparis micracantha* DC. and *Gnetum montanum* Markgr. are becoming rare because of overexploitation without sustainability. On the other hand, the result of this study was not in agreement with other studies which reported that leaves [9, 10], the whole plant [11] and the stem [12] were the most used parts.

### Tastes of individual medicinal plants

Tastes of individual medicinal plants play a key role for plant selection when used as medicine in the traditional Thai medicine system. Different tastes are associated with curing properties and applications. Based on traditional Thai medicine concepts, tastes are divided into ten types i.e., astringent, sweet, nauseating, bitter, hot, fat, salty, sour, aromatic and bland (tasteless). The idea of taste is in agreement with the Ayurvedic system [13] and traditional uses of plants by tribal communities [14, 15]. The result of this study showed that all medicinal plants were classified into nine groups by their tastes (Table 2). The most frequently found taste was bland (20 species) followed by bitter (18 species) and aromatic and nauseating (16 species each). Bland plants were clearly found to be used for fever (six species) and for their diuretic properties (four species). They were generally used as ingredients together with bitter plants for making remedies for the treatment of fever. Traditional healers explained that bitter plants can kill pathogens while bland plants decrease body temperature by urine excretion. The result of the study was not accordant with Ankli [14], who reported that Maya of the Yucatan peninsula (Mexico) used bland plants (tasteless) for women’s diseases whereas bitter plants were used to treat animal bites and dermatological conditions. Aromatic and hot plants were generally used to treat gastrointestinal diseases (16 species and 12 species, respectively), especially flatulence. Flatulence was the most prominent symptom that occurred in most people, therefore it has various medicinal plants for treatment. Likewise, Ankli [14] reported that aromatic plants were used to treat gastrointestinal diseases. Nauseating plants were related with various illnesses such as gastrointestinal diseases (six species), skin conditions (seven species), muscle pain (two species), women’s diseases and bleeding (one species each).

**Table 2:** Medicinal plant tastes and medicinal applications (cited by more than five traditional healers)

Groups of ailment	Ailment	Aromatic	Astringent	Bitter	Bland	Hot	Nauseating	Sour	Sweet	Fat	Total
Gastrointestinal system	flatulence	11				4					15
(34 species)	canker				1						1
	peptic ulcer		1			1					2
	intestinal infections		1	1			1				3
	laxative, constipation			2		1	2	2	1		8
	hemorrhoids						2				2
	expel worms						1				1
	toothache	1				1					2
Respiratory system (6 species)	eliminate sputum, cough, sore throat			2				3	1		6
Fever, malaria (19 species)				12	6					1	19
Skin conditions	eczema, scabies, abscess		2				6				8
(12 species)	animal bites, itching				2						2
	skin swelling, burns			1	1						2
	stop bleeding						1				1
Female problems (5 species)	women’s diseases				1	2	1			1	5
Antidote (2 species)					2						2
Musculoskeletal system (8 species)			1		3	1	2			1	8
Nephrological system (4 species)	diuretic				4						4
The heart and circulatory system	dizziness, tonifies heart	3									3
(3 species)											
Miscellaneous (4 species)	improve body elements	1				2				1	4
	total	16	5	18	20	12	16	5	2	4	98

The most interesting group was the one related to skin conditions. Skin conditions were treated using a combination of nauseating, astringent, bland and bitter plants. The preferred treatment for various infections used nauseating and astringent tasting plants, whereas the preferred option for swellings was bitter and bland plants. In the case of chronic skin diseases, all four tastes of plants were combined to make remedies. Astringent plants were also used to treat intestinal infections such as dysentery, diarrhea and peptic ulcer. This result was similar to the study of Leonti [16], which revealed that diarrhea and dysentery were treated with astringent plants, for example pericarp of *Garcinia mangostana* L., an astringent plant which is used for its anti-inflammatory activity [17] and extracts of *Garcinia mangostana* L. and *Punica granatum* L. which exhibit antibacterial activity [18]. *Curcuma longa* L., an astringent plant, has long been used in traditional medicine and as a food additive. Its rhizome has played a key role in traditional medicine for treating various diseases such as flatulence, peptic ulcer, intestinal infection, and skin conditions. It was scientifically confirmed in various aspects such as antioxidant activity [19, 20], anti-inflammation [19, 21], antibacterial activity [22] and anti-ulcer activity [23]. The curing properties of other tastes are represented in Table 2. However, this study suggested that the selection of medicinal plants to be used as medicines and the efficacy of treatments derives from a combination of the experience of individual healers as well as medicinal plant selection. This study was in agreement with Casagrande [15] who reported that taste should not be allowed to predict the properties of medicinal plants of the Tzeltal Maya alone, but it should be combined with the experience of each traditional healer. However, Ankli [14] mentioned that taste and secondary products of medicinal plants are relative and traditional knowledge can help healers to distinguish between medicinal and non-medicinal plants.

A greater understanding of the medicinal plant tasting concept is necessary for medicinal plant selection so that more efficient traditional prescriptions can be made. Additionally, the relationship between plant tastes and biological activities have been scientifically confirmed (Table 3). Some common medicinal plants have been used for a long time in traditional medicine such as *Curcuma longa* L.; it is used for wound healing, peptic ulcer and various skin diseases. These activities were already confirmed by scientific validation and were accordant with traditional uses, including other medicinal plant species such as *Aloe vera* (L.) Burm. f., *Amomum testaceum* Ridl., *Andrographis paniculata* (Burm. f.) Wall. ex Nees, *Boesenbergia rotunda* (L.) Mansf., *Centella asiatica* (L.) Urb., *Cissus quadrangularis* L., *Eurycoma longifolia* Jack, *Punica granatum* L., *Solanum trilobatum* L., *Thunbergia laurifolia* Lindl., *Tiliacora trianda* (Colebr.) Diels, *Zingiber montanum* (Koenig) Link ex Dietr. and *Zingiber officinale* Roscoe. These have all been scientifically confirmed.

**Table 3:** The relationship between ailment and taste of some medicinal plant species

Ailment	Tastes of plant species	Example	Pharmacological activity
Gastrointestinal system			
- flatulence, peptic ulcer	hot, aromatic	*Acorus calamus* L.	anti-ulcer activity [43]
		*Boesenbergia rotunda* (L.) Mansf.	anti-ulcerogenic property [44]
		*Cyperus rotundus* L.	cytoprotective effects [45]
		*Kaempferia galanga* L.	against gastric mucosal [46]
		*Kaempferia parviflora* Wall. ex. Baker	anti-*Helicobacter pylori* [47]
		*Ocimum basilicum* L.	anti-ulcer activity [48]
		*Ocimum tenuiflorum* L.	antimicrobial activity [49]
		*Zingiber officinale* Roscoe	anti-ulcerogenic effect [50]
- intestinal infections	astringent	*Curcuma longa* L.	antioxidant and anti-inflammatory activities [51], antibacterial activity [22] antibacterial and mycobacterial activities [20]
		*Curcuma zedoaria* (Berg) Roscoe	antimicrobial activity [51], anti-inflammation [52]
- diarrhea, dysentery	bitter	*Zingiber zerumbet* (L.) Sm.	antimicrobial activity [53], antinociceptive [54]
	astringent	*Punica granatum* L.	antidiarrheal activity [55], anti-inflammatory effect [56]
- laxative, constipation	sour	*Tamarindus indica* L.	antioxidative effect [57]
	sweet	*Cassia fistula* L.	pediatric functional constipation [58], laxative effect [59]
	hot	*Croton tiglium* L.	gastrointestinal effect [59]
- hemorrhoids	nauseating	*Cissus quadrangularis* L.	efficacy and side effects of acute hemorrhoids [60] analgesic and anti-inflammatory activities [61]
- toothache	hot	*Zanthoxylum nitidum* (Roxb.) DC.	anti-inflammation [62, 63] antibacterial activity [62], analgesic activity [64]
Fever, malaria	bitter	*Andrographis paniculata* (Burm. f.) Wall. ex Nees	antimalarial activity [65], antibacterial activity [66], anti-inflammation [67], antiviral activity [68]
		*Brucea javanica* (L.) Merr.	antimalarial activity [69], antiplasmodial activity [70]
		*Centella asiatica* (L.) Urb.	Antinociceptive and anti-inflammatory activity [71], anti-allergic activity [72]
		*Clerodendrum petasites* S. Moore	antipyretic activity [61]
		*Eurycoma longifolia* Jack	antiplasmodial activity [73], antiparasitic activity [74]
		*Momordica charantia* L.	Antibacterial and antifungal activities [75]
		*Phyllanthus urinaria* L.	anti-inflammation [76], anti-HSV [77]
		*Tinospora crispa crispa* (L.) Miers ex Hook. f. & Thomson	antimalarial activity [78], antinociceptive and anti-inflammatory activities [79]
	bland	*Tiliacora trianda* (Colebr.) Diels	antimycobacterial activity [80]
		*Vernonia cinerea* (L. ) Less.	antipyretic and anti-inflammatory activities [81], antibacterial activity [82], antimalarial activity [83]
Skin conditions			
- eczema, scabies, abscess itching, measles, skin diseases, poisonous animal bites,	nauseating	*Amorphophallus paeoniifolius* (Dennst.) Nicolson	anti-inflammatory activity [84]
		*Datura metel* L.	antimicrobial activity [85], antimycotic activity [86]
		*Mitragyna speciosa* (Roxb.) Korth.	antioxidant and antibacterial activities [87], anti-inflammatory and antinociceptive activities [88]
		*Rhinacanthus nasutus* (L.) Kurz	antimicrobial activity [89], antiallergic activity [90], antifungal and anti-inflammatory activities [91]
		*Senna alata* (L. ) Roxb.	antiallergic activity [92], antifungal activity [93]
		*Senna tora* (L. ) Roxb.	antioxidant and antibacterial activities [94] antibacterial activity
		*Stemona tuberosa* Lour.	antibacterial activity [95]
- burns, skin swelling	bland	*Aloe vera* (L.) Burn. f.	antioxidant and anti-inflammatory activities [96] wound healing property [97]
- stop bleeding	nauseating	*Chromolaena odoratum* (L.) R. M. King & H. Roxb.	hemostatic and wound healing properties [98]
Respiratory system			
- eliminates sputum, cough,	sour	*Abrus precatorius* L.	anti-inflammatory and antiallergic activity [99]
sore throat		*Albizia myriophylla* Benth.	antibacterial activity [100]
		*Phyllanthus emblica* L.	anti-inflammatory activity [101], anti-Coxsackie Virus B3 [102]
		*Tamarindus indica* L.	antimicrobial activity [103]
	bitter	*Solanum trilobatum* L.	antimicrobial activity [104], anti-inflammatory and analgesic activities [105]
Female problems	fat	*Sida rhombifolia* L.	antinociceptive and anti-inflammatory activities [106]
Nephrological system			
- diuretic	bland	*Orthosiphon aristatus* (Blume) Miq.c	anti-inflammatory activity [107]
		*Pluchea indica* (L. ) Less.	anti-inflammatory activity [108], antimicrobial activity [109]

However, there are many medicinal plants which have been commonly used in the area. The activities of these medicinal plants which are accordant with traditional uses have been unproven such as, *Aquilaria malaccensis* Lam., *Baliospermum solanifolium* (Burm.) Suresh, *Bridelia ovata* Decne., *Cardiospermum halicacabum* L., *Capparis micracantha* DC., *Clerodendrum serratum* (L.) Moon, *Donax grandis* (Miq.) Ridl., *Drynaria quercifolia* (L.) Sm., *Excoecaria oppositifolia* Griff., *Fagraea fragrans* Roxb., *Gnetum montanum* Markgr., *Goniothalamus macrophyllus* (Blume) Hook. f. & Thomson, *Gymnopetalum chinense* (Lour.) Merr., *Lasia spinosa* (L.) Thwaites, *Lepionurus sylvestris* Blume, *Panicum repens* L., *Saprosma brunneum* Craib, *Schumannianthus dichotomus* (Roxb.) Gagnep. and *Shirakiopsis indica* (Willd.) Esser.

### Groups of ailments and medicinal plants

**Fig. 6:**
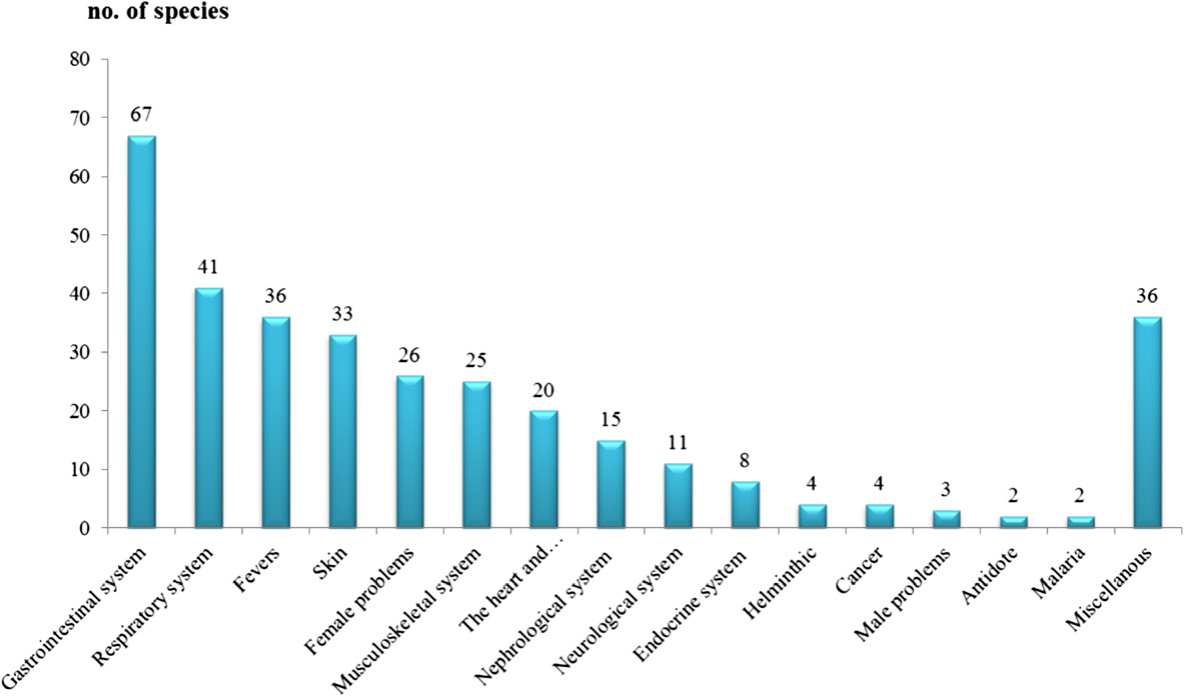
Groups of ailments

One hundred and fifty one medicinal plants were divided into 16 ailment groups (Fig. 6). The largest number of medicinal plants were found to be used for treating the gastrointestinal system, such as flatulence, toothache, canker, stomachache, constipation, diarrhea, dysentery, peptic ulcer and liver diseases, for which 67 species were used, for example *Zingiber zerumbet* (L.) Sm. for flatulence and dysentery, *Baliospermum solanifolium* (Burm.) Suresh for hemorrhoids and constipation and *Clerodendrum serratum* (L.) Moon for hemorrhoids. Neamsuvan [24] mentioned that gastrointestinal disorders were frequently found in southern Thailand because of climate and food consumption culture and also reported that *Senna alata* (L.) Roxb. was predominantly used for constipation, which was in agreement with the present study. The second largest group of ailments was found to be with the respiratory system, such as asthma, elimination of sputum, coughs, sore throats and sinusitis, for which 41 species were used, for example *Justicia adhatoda* L. for the elimination of sputum and coughs, *Millingtonia hortensis* L. f. for sinusitis and asthma. The third largest group of ailments was fever for which 36 species of plants were found, such as *Capparis micracantha* DC., *Clerodendrum petasites* (Lour.) S. Moore, *Harrisonia perforata* (Blanco) Merr. and *Tiliacora trianda* (Colebr.) Diels. These plants constitute the ingredients of a traditional Thai formula which is called “Ya-Ha-Rak” and it has been routinely used for fever treatment. Furthermore, *Harrisonia perforata* (Blanco) Merr. was reported that its aqueous extract showed the highest activity against HIV-1 IN [25]. Other interesting plant species for the treatment of fever include *Andrographis paniculata* (Burm. f.) Wall. ex Nees, *Donax grandis* (Miq.) Ridl., *Gymnopetalum chinense* (Lour.) Merr., *Panicum repens* L., *Tinospora crispa* (L.) Miers ex Hook. f. & Thomson and *Vernonia cinerea* (L.) Less. Interestingly, these plants are bitter and bland. According to the belief of traditional Thai healers, bitter plants have the potential to reduce body temperature and kill microorganisms, whereas bland plants are used as diuretics which decrease body heat. In Sating Phra, peninsular Thailand, *Vernonia cinerea* (L.) Less was used for wound healing [26]. This particular usage differed from examples in this study. Additionally, *Vernonia cinerea* (L.) Less was well known for smoking cessation. Interestingly, *Chromolaena odoratum* (L.) R. M. King & H. Roxb. was commonly used for wound bleeding. [26–29]. The study of Pandith [30] confirmed that hemostatic and wound healing activities were related with the expression of genes, heme oxygenase-1, thromboxane synthase and MMP-9. The smallest number of medicinal plants were used to treat malaria and as antidotes. For malaria treatment, two plant species were found, i.e., *Brucea javanica* (L.) Merr. and *Eurycoma longifolia* Jack. The taste of both species is bitter. Based on traditional Thai medicine, malaria is a type of fever which is frequently treated with bitter medicinal plants. Maneenoon [31] reported that *Eurycoma longifolia* Jack was used by the Sakai tribe, a minority of southern Thailand, to treat fevers as well as malaria. In northern Thailand, the whole plant of *Brucea javanica* (L.) Merr was used to treat itching, whereas malaria was treated with *Phyllanthus urinaria* L. [27]. As recorded here *Phyllanthus urinaria* L. was used for treating normal fever or body pain caused by fever. *Tiliacora trianda* (Colebr.) Diels and *Thunbergia laurifolia* L. were used for their antidotal properties. Both species have been widely used by Thai traditional healers for treating food poisoning and environmental toxicants. Furthermore, *Tiliacora trianda* (Colebr.) Diels has been added to traditional Thai formulas to lessen the toxicity of the formula. Similarly, *Thunbergia laurifolia* L. was widely used as an antidote by Tai Yai [27], Karen [29] and Buddhist and Muslim Thais in southern Thailand [32]. Pharmacological activities of both plants have been scientifically confirmed, especially the properties of *Thunbergia laurifolia* Lindl., which has been proven effective in the detoxification of insecticide residues [33]. It is used for its antimutagenic activity [34], in the treatment of drug addiction [35], for its antioxidant activity and in the detoxification of cytotoxicity [36], against Pb(NO_3_)_2_ toxicity in Nile tilapia [37], against chronic toxicity [38], for protection against oxidative stress and cell death in brain tissues caused by lead exposure [39] and for prevention of renal toxicity induced by cadmium [40].

The accepted use of *Tiliacora trianda* (Colebr.) Diels is as a detoxifying agent as well as a fever reducing treatment. The result was in agreement with Upho [32], which indicated that this plant species was used as a refrigerant. Saiin & Markmee [41], reported that the extracts of *Tiliacora trianda* (Colebr.) Diels and *Harrisonia perforata* (Blanco) Merr. inhibited *Plasmodium falciparum*, which is the cause of malaria. Moreover, Sireeratawong [42] mentioned that the water extracts of *Tiliacora trianda* (Colebr.) Diels did not cause acute or subchronic toxicities in rats. Based on traditional uses, the consumption of leaf juice of *Tiliacora trianda* (Colebr.) Diels decreases body temperature, but it must not be consumed continuously for longer than seven days.

### Interesting medicinal plants that are promoted to cure common diseases in rural areas and new properties of some medicinal plants

Many medicinal plants and traditional prescriptions referred to in the present study have been strongly promoted in rural areas for self-healing. Their healing properties, as confirmed by scientific approach, have proven to be consistent. For example, *Thunbergia laurifolia* L. is strongly promoted for chemical detoxification in agriculturist. *Andrographis paniculata* (Burm. f.) Wall. ex Nees, *Phyllanthus urinaria* L., Ya-Ha-Rak (composed of five roots of *Harrisonia perforata* (Blanco) Merr., *Capparis micracantha* DC., *Tiliacora triandra* (Colebr.) Diels, *Clerodendrum petasites* (Lour.) S. Moore and *Ficus racemosa* L.) and Tri-Pha-La (composed of fruits of *Phyllanthus emblica* L., *Terminalia chebula* Retz. and *Terminalia bellirica* (Gaertn.) Roxb.) are used for treating fever, especially Tri-Pha-La, which is widely used not only for reducing fever but also for tonifying the body elements. Pulp of *Cassia fistula* L. is mixed with bitter salt and Ya-Dam (latex of *Aloe vera* L.) to increase the efficacy for the treatment of constipation. *Clinacanthus nutans* (Burm. f.) Lindau, *Garcinia mangostana* L., *Rhinacanthus nasutus* (L.) Kurz and *Senna alata* (L.) Roxb. are appropriate for treating skin disorders, and many members of Zingiberaceae are suitable for gastrointestinal disorders. Local health care compliance officers should promote these plants to rural people, including advice on their planting and conservation in local communities. The main advantages which rural people gain from these plants are reduced expenses and the avoidance of side effects from chemical drugs.

The present study found new properties of *Ocimum tenuiflorum* L. and *Zingiber montanum* (Koenig) Link ex Dietr. Normally, both plants are well known for treating flatulence. In addition, the latter is widely used for the treatment of muscle pain. Furthermore, the leaves of *Ocimum tenuiflorum* L. are crushed and the extract is anointed on the chest and back of children for the treatment of asthma. An extract of rhizome of *Zingiber montanum* (Koenig) Link ex Dietr is mixed with Ya-Dam and placed on swollen areas for the treatment of bruises.

## Conclusions

The study concluded that even though conventional medicine is available, many people in rural communities still continue to depend on traditional Thai medicine, and highly experienced traditional healers are still important to the communities. Unfortunately, the rapid disappearance of traditional medicine and natural resources due to urbanization suggests that unrecorded data may be lost forever. Therefore, further study will be needed for systematic documentation of traditional Thai medicine including scientific confirmation through biological activities. Clinical studies will also be required.
